# High-Resolution Estimates of Crossover and Noncrossover Recombination from a Captive Baboon Colony

**DOI:** 10.1093/gbe/evac040

**Published:** 2022-03-24

**Authors:** Jeffrey D. Wall, Jacqueline A. Robinson, Laura A. Cox

**Affiliations:** 1 Institute for Human Genetics, University of California San Francisco, USA; 2 Center for Precision Medicine, Department of Internal Medicine, Wake Forest School of Medicine, Winston-Salem, USA

**Keywords:** recombination, nonhuman primates, noncrossovers, linkage disequilibrium

## Abstract

Homologous recombination has been extensively studied in humans and a handful of model organisms. Much less is known about recombination in other species, including nonhuman primates. Here, we present a study of crossovers (COs) and noncrossover (NCO) recombination in olive baboons (*Papio anubis*) from two pedigrees containing a total of 20 paternal and 17 maternal meioses, and compare these results to linkage disequilibrium (LD) based recombination estimates from 36 unrelated olive baboons. We demonstrate how COs, combined with LD-based recombination estimates, can be used to identify genome assembly errors. We also quantify sex-specific differences in recombination rates, including elevated male CO and reduced female CO rates near telomeres. Finally, we add to the increasing body of evidence suggesting that while most NCO recombination tracts in mammals are short (e.g., <500 bp), there is a non-negligible fraction of longer (e.g., >1 kb) NCO tracts. For NCO tracts shorter than 10 kb, we fit a mixture of two (truncated) geometric distributions model to the NCO tract length distribution and estimate that >99% of all NCO tracts are very short (mean 24 bp), but the remaining tracts can be quite long (mean 4.3 kb). A single geometric distribution model for NCO tract lengths is incompatible with the data, suggesting that LD-based methods for estimating NCO recombination rates that make this assumption may need to be modified.

**Significance**

Most homologous recombination events are noncrossovers (NCOs), but little is known about NCO conversion tract lengths. Here, we utilize whole-genome sequence data from large baboon pedigrees to estimate the NCO tract length distribution and to study other aspects of recombination.

## Introduction

Homologous recombination is a fundamental biological process, thought to be necessary for the proper segregation of chromosomes during meiosis and essential for the efficacy of natural selection. Recombination rates in higher eukaryotes are generally measured using (1) genetic comparisons between parents and offspring (e.g., using genotype or sequence data), (2) genotyping or sequencing of single or pooled sperm (i.e., potential gametes), or (3) indirect estimation via statistical methods that quantify the relationship between linkage disequilibrium (LD) and recombination. Each of these three approaches involves tradeoffs regarding cost/effort and the breadth and depth of information they can provide. In particular, only pedigree-based studies provide both sex-specific recombination estimates and direct identification of both crossover (CO) and noncrossover (NCO) recombination events, but they are more difficult to conduct due to sample acquisition challenges.

Recombination is thought to arise from double-strand breaks (DSBs) that occur after the pairing of homologous chromosomes during meiosis. Depending on how these breaks are resolved, the result can either be CO recombination, which involves the reciprocal transfer of large chromosomal regions between homologs, and NCO recombination (colloquially called “gene conversion”), involving the non-reciprocal replacement of short tracts of DNA from one homolog to another (Orr-Weaver et al. 1981; Szostak et al. 1983). Since COs are also associated with gene conversion tracts at the DSB location, we will use the term NCO recombination to describe homologous gene conversion not associated with a nearby CO.

Theory predicts a close relationship between recombination and patterns of LD since homologous recombination will tend to shuffle haplotypes and break down allelic associations. Population genetic analyses of dense genotype and sequence data, along with sperm typing studies, have shown that most human COs happen in narrow (1–2 kb) “hotspots” (Chakravarti et al. 1984; Jeffreys et al. 2001; Crawford et al. 2004; Myers et al. 2005), and that this fine-scale structuring of recombination rates can help explain the block-like structure of LD in many parts of the genome (Wall and Pritchard 2003). In most vertebrates, the locations of these hotspots are mediated by the zinc finger *PRDM9* (reviewed in Paigen and Petkov 2018), and recombination hotspot locations are generally not shared across closely related species (Ptak et al. 2005; Auton et al. 2012; Stevison et al. 2016).

Much less is known about NCO recombination in species where only one of four meiotic products can be recovered (i.e., species where gene conversion must be inferred rather than directly observed). A handful of studies in humans and model organisms have estimated that most recombination events are NCOs, but mean tract lengths are quite short—tens or hundreds of base pairs (Jeffreys and May 2004; Baudat and de Massy 2007; Cole et al 2010; Comeron et al. 2012; Wijnker et al. 2013; Li et al. 2019). This short tract length makes NCO recombination especially difficult to study. In particular, for species with low levels of heterozygosity (e.g., most mammals), many NCO tracts are undetectable because the donor and converted sequences are identical. In most of the remainder, only a single heterozygous site is converted, making NCO recombination difficult to distinguish from simple genotype/sequencing errors. While statistical methods have been developed for estimating NCO recombination parameters indirectly from segregating patterns of genetic variation (Frisse et al. 2001; Gay et al. 2007; Yin et al. 2009; Padhukasahasram and Rannala 2013), these methods are not very accurate primarily because of the small/negligible effect that most NCO tracts have on patterns of genetic variation. In addition, these methods generally assume that NCO tract lengths follow a geometric distribution, which may not be biologically realistic. Because of this, studies of NCO recombination have typically focused on identifying events by comparing the patterns of genetic inheritance of offspring (or potential offspring in the case of sperm typing) from their parents (Jeffreys and May 2004; Comeron et al. 2012; Wijnker et al. 2013; Williams et al. 2015; Halldorsson et al. 2016; Li et al. 2019).

Among mammals, NCO recombination has been most studied in humans, with several sperm typing studies (Jeffreys and May 2004; Jeffreys and Neumann 2005; Webb et al. 2008; Odenthal-Hesse et al. 2014), two large pedigree-based studies (Williams et al. 2015; Halldorsson et al. 2016), and a study of genetic variation in autozygous tracts of consanguineous individuals (Narasimhan et al. 2017). Two observations from these studies stand out. First, both pedigree-based studies found evidence for complex NCO events, involving multiple noncontiguous gene conversion tracts that are physically near each other, from the same meiosis, and not associated with a nearby CO (Williams et al. 2015; Halldorsson et al. 2016). Second, both studies also found evidence for apparent long (i.e., >20 kb), contiguous NCO tracts. If real, these long tracts are suggestive of a separate molecular mechanism distinct from the gene conversion expected under the standard DSB model. It is possible, though, that they reflect a rare CO interference-independent recombination process, or that they are actually complex NCO events with smaller tract sizes that are miscalled due to low marker density.

**Fig. 1.**
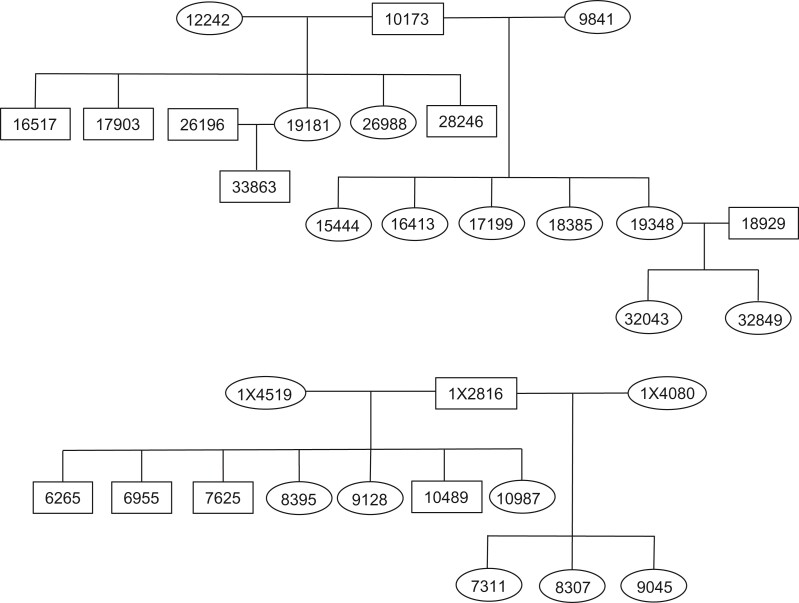
Schematics of the two baboon pedigrees used in this study.

In this study, we examine patterns of recombination with a focus on NCO tracts using olive baboons (*Papio anubis*) in the colony housed at the Southwest National Primate Research Center (SNPRC). We generate and analyze high-coverage whole-genome sequence data from two pedigrees with large sib-ships (fig. 1), which allows us to estimate sex-specific recombination rates, identify NCO recombination events, and evaluate the long-range accuracy of the current Panubis1.0 genome assembly (cf. Batra et al. 2020). This assembly used Hi-C contact data to join contigs into scaffolds, and the low-resolution linkage map we generate here allows us to assess the accuracy of this approach.

Our choice of baboons was motivated in part by the availability of an extremely large pedigreed colony at the SNPRC, as well as the higher levels of diversity found in baboons relative to humans (e.g., Robinson et al. 2019). Our expectation is that the increased marker density will provide greater resolution on the size distribution of NCO tracts and that our study of a nonhuman primate will help elucidate whether some of the specific recombination patterns observed in humans can be generalized to a wider group of species.

## Results

### Baboon Genetic Map

We identified COs and NCO recombination events from a total of 20 paternal and 17 maternal meioses (supplementary table S1, Supplementary material online). In total, we identified 842 autosomal COs with a median resolution (i.e., the size of the region over which the CO location could be placed) of 7.7 kb. This corresponds to a sex-averaged autosomal genetic map length of 2,293 cM (2,080 cM in males and 2,506 cM in females). Our estimate is 16% larger than a previous estimate based on microsatellite data (Rogers et al. 2000), which reflects both the longer and more complete baboon genome assembly that we used and the much greater marker density of our study. Overall, our results are consistent with the growing body of evidence suggesting that Old World monkeys have shorter genetic map lengths, as measured by direct identification of COs in pedigrees, than do humans and great apes (e.g., Broman et al. 1998; Rogers et al. 2000, 2006; Kong et al. 2002; Jasinska et al. 2007; Venn et al. 2014).

We also estimated local recombination rates from patterns of LD in 36 unrelated olive baboons using pyrho (Spence and Song 2019). We found that rate estimates are significantly higher within distal regions (≤10 Mb from chromosome ends) relative to proximal regions (>10 Mb from chromosome ends) (two-sided Mann–Whitney *U* test, *P* < 2.2 × 10^−16^, supplementary fig. 1, Supplementary material online).

### Identifying Potential Genome Assembly Errors

**Fig. 2.**
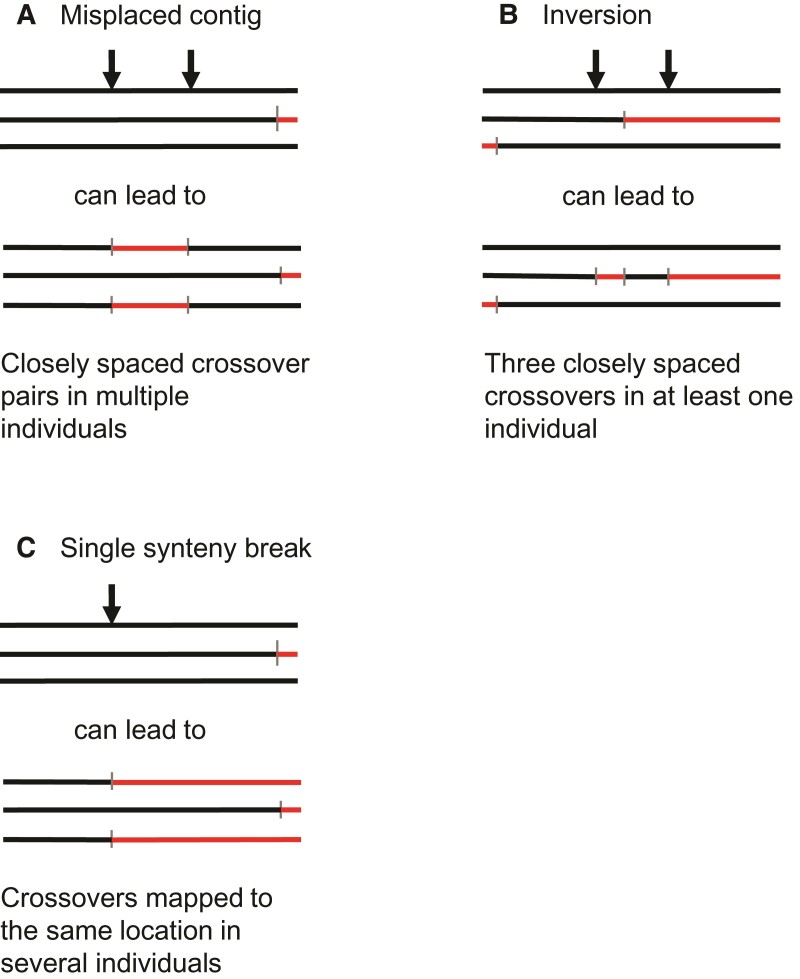
Detecting assembly errors from abnormal CO patterns. Horizontal lines represent homologous chromosomes in different individuals, arrows represent the locations of synteny breaks caused by different assembly errors, and vertical hashmarks represent the locations of COs. Chromosomes are colored in alternating red and black, representing which of the two parental chromosomes is inferred to be ancestral. Note that synteny breaks result in spuriously inferred COs only in some individuals (but not others). (*A*) Misplaced contig, (*B*) inversion, and (*C*) single synteny break.

**Fig. 3.**
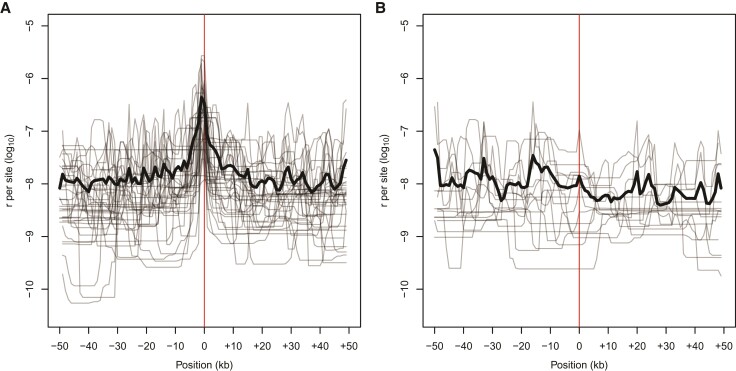
pyrho-based estimates of recombination rate from patterns of LD at (*A*) breaks of synteny in the current baboon assembly identified from abnormal CO patterns (*n* = 35), and (*B*) Selected regions where the Panubis1.0 assembly has been “corrected” (*n* = 15). Lighter lines show estimates of the local recombination rates from 50 kb regions up- and down-stream of the focal point (location of a synteny break or assembly correction, indicated by the red line), and the bold line shows the average. In (*A*), the peak centered at 0 shows elevated recombination rates around putative breaks in synteny. In (*B*), the lack of such a peak centered at 0 shows the impact of correcting the underlying assembly.

In performing quality control for our genetic map, we identified several abnormal apparent CO patterns that likely reflect errors in the Panubis1.0 genome assembly (fig. 2). These included a total of 16 potential inversions, 3 misplaced contigs, and 1 potential translocation (supplementary table S2, Supplementary material online). We then used LD-based estimates of recombination using pyrho (Spence and Song 2019) to examine whether patterns of LD provided any additional support. On average, estimated recombination rates at putative synteny breaks are roughly 20 times higher than the estimated rates in the flanking sequences (fig. 3*A*), consistent with the decrease in LD expected across genome assembly error breakpoints. For six proposed inversions and the translocation (supplementary table S2, Supplementary material online), pyrho estimates provide corroborating evidence in finding low levels of estimated recombination (i.e., evidence for synteny) across the “corrected” breakpoints (fig. 3*B*).

### NCO Recombination

After stringent filtering, we identified a total of 325 bi-allelic SNPs contained in 263 tracts (supplementary table S3, Supplementary material online) that were inferred to be converted due to NCO recombination in tracts <10 kb in length. (See below for a discussion of why we exclude longer NCO tracts.) Of the 39 events involving the conversion of more than one heterozygote, the minimal length of the inferred NCO tract was generally small (median = 42 bp) but had a long tail of occasionally longer tracts (mean = 167 bp, including 10 tracts longer than 1 kb).

Overall, we estimated a sex-averaged NCO rate of 7.52 × 10^−6^ per site per generation (paternal NCO rate = 5.34 × 10^−6^ and maternal NCO rate = 9.71 × 10^−6^), roughly comparable to NCO rate estimates in humans (e.g., Williams et al. 2015; Halldorsson et al. 2016; Narasimhan et al. 2017). As with previous human studies (Williams et al. 2015; Halldorsson et al. 2016), we found a handful of more complex NCO recombination events, including seven regions containing multiple non-contiguous NCO tracts and nine NCO regions associated with a nearby CO (supplementary table S3, Supplementary material online; note that three regions involve non-contiguous NCO tracts that are also associated with a nearby CO).

In addition, we identified 10 regions consistent with a potential NCO tract of length 10–100 kb (Table 1). Of these, six were also identified as potential inversion errors in the underlying genome assembly, and three others overlapped with non-inversion potential genome assembly errors (supplementary table S2, Supplementary material online). In summary, nine out of ten potential long NCO tracts are consistent with being artifacts caused by problems with the Panubis1.0 genome assembly, suggesting that long NCO tracts are difficult to accurately identify in baboons. If we include the remaining long NCO tract into the rate calculation, the estimated sex-averaged NCO rate increases to 8.01 × 10^−6^ per site per generation (paternal NCO rate = 5.34 × 10^−6^ and maternal NCO rate = 1.07 × 10^−5^). However, we focus on tracts <10 kb for all subsequent analyses.

**Table 1:** List of Apparent NCO Tracts Longer Than 10 kb.

Chrom.	Parent	Offspring	Minimum tract length	# of NCO sites	Overlap with inversion or break of synteny?
3	9841	19,348	40,934	22	No
4	9841	15,444	24,809	39	Yes
6	10,173	18,385	55,304	62	Yes
7	10,173	15,444	39,253	2	Yes
7	12,242	26,988	45,787	22	Yes
8	12,242	28,246	61,766	9	Yes
11	1X2816	10,489	24,481	4	Yes
13	1X2816	8307	84,614	54	Yes
13	10,173	16,517	86,445	48	Yes
16	12,242	17,903	12,403	19	Yes

### GC Bias of NCO Tracts

GC-biased gene conversion (gBGC) is a selectively neutral process whereby gene conversion events containing an AT/GC heterozygote in the parent are preferentially resolved to contain the G or C allele in the gamete (Galtier and Duret 2007; Duret and Galtier 2009). Both sperm typing studies (Odenthal-Hesse et al. 2014) and pedigree-based studies (Williams et al. 2015; Halldorsson et al. 2016) in humans have quantified the strength of gBGC in humans. Of the 224 NCO tracts that were informative on gBGC in our study, 129 of them (57.6%) show a transmission bias toward G or C alleles. While this proportion is significantly more than 50% (*P* = 0.014, one-tailed binomial test), it is also significantly less than (*P* = 6.8 × 10^−4^, one-tailed binomial test) the 68% GC bias estimated from human pedigree studies (Williams et al. 2015; Halldorsson et al. 2016).

### Age versus Recombination Rate

Previous human recombination studies have documented increases in both CO rate (Kong et al. 2004; Martin et al. 2015) and NCO rate (Halldorsson et al. 2016) with increasing maternal age. While we are underpowered to detect any true correlations between recombination rate and parental age, we did find a marginally significant association between NCO rate and paternal age (*P* = .036; raw data in supplementary table S3, Supplementary material online). All other comparisons of CO or NCO rate with paternal or maternal age were not significant (*P* > 0.1).

### Regional Variation in Recombination Rates

**Fig. 4.**
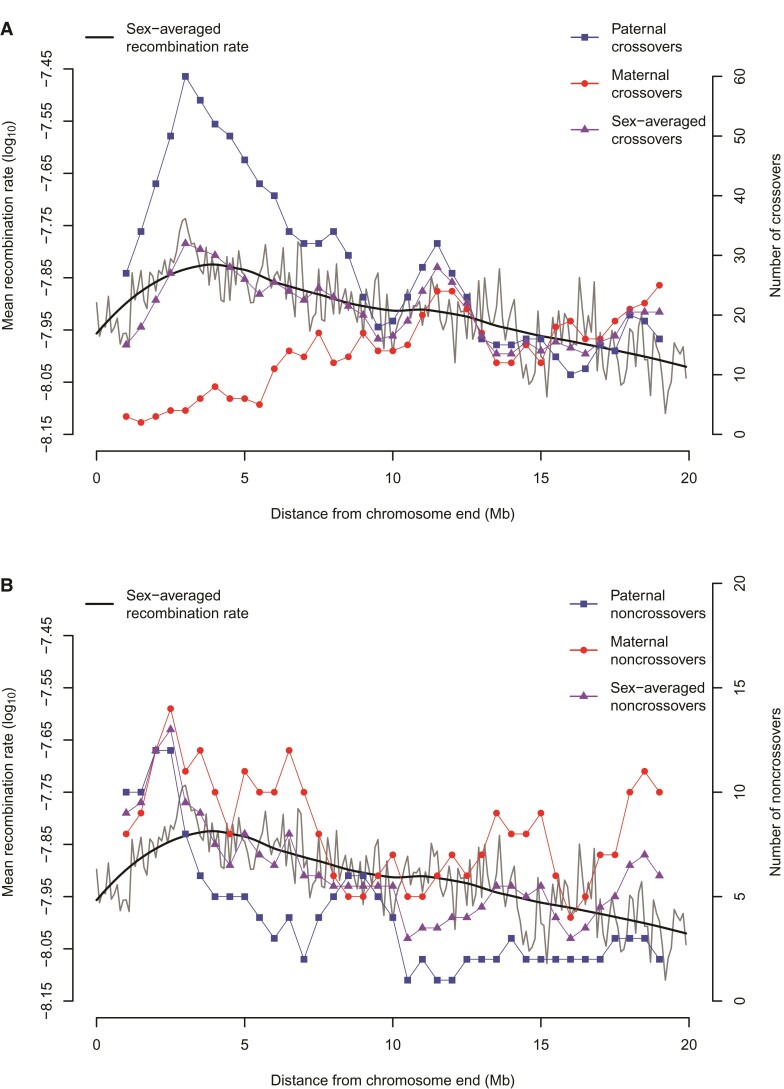
Recombination rates as a function of distance from telomeres. Comparison of LD-based recombination estimates from pyrho (gray and black) with paternal (blue) and maternal (red) (*A*) CO counts, and (*B*) NCO recombination counts. The thin black line shows pyrho-based recombination rates in non-overlapping 100 kb windows, and the bold black line shows a smoothed local regression (loess with span 0.5). CO and NCO counts represent rolling means estimated from 2 Mb windows with a 500 kb step size.

**Fig. 5.**
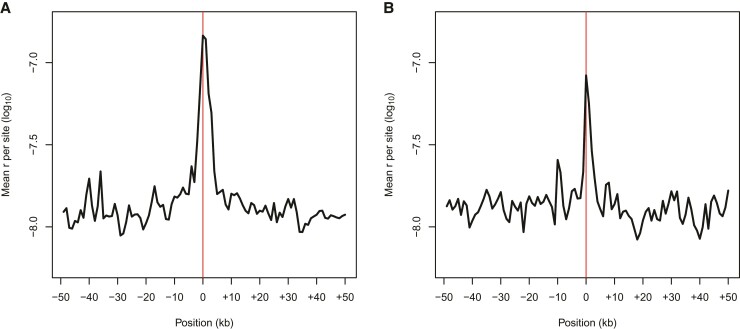
Elevation of pyrho recombination rate estimates near the sites of (*A*) CO (*n* = 328) and (*B*) NCO (*n* = 259) events. Plots show the average of pyrho-based recombination rate estimates for 50 kb regions up- and down-stream of putative CO and NCO locations identified using information from the pedigrees.

We tabulated the relative numbers of CO and NCO recombination events as a function of distance from telomeres and stratified the results by sex. We then compared these with sex-averaged recombination rate estimates based on patterns of LD (fig. 4). As with humans, we find that the male/female CO ratio is higher in distal regions and lower in proximal regions further from the chromosome ends. Near baboon telomeres, males have significantly higher CO rates (compared with male CO rates across the rest of the genome) *and* females have significantly lower CO rates (compared with female CO rates across the rest of the genome) (fig. 4*A*). We observe a significantly higher male NCO rate near the ends of chromosomes as well (fig. 4*B*), but did not observe any correlation between female NCO rate and chromosome ends or between CO rate and NCO rate. Consistent with a (partial) decoupling of local CO and NCO rates, we find that pyrho recombination rate estimates are higher near inferred CO locations (fig. 5*A*) than near inferred NCO locations (fig. 5*B*).

### NCO Tract Length Distribution

**Fig. 6.**
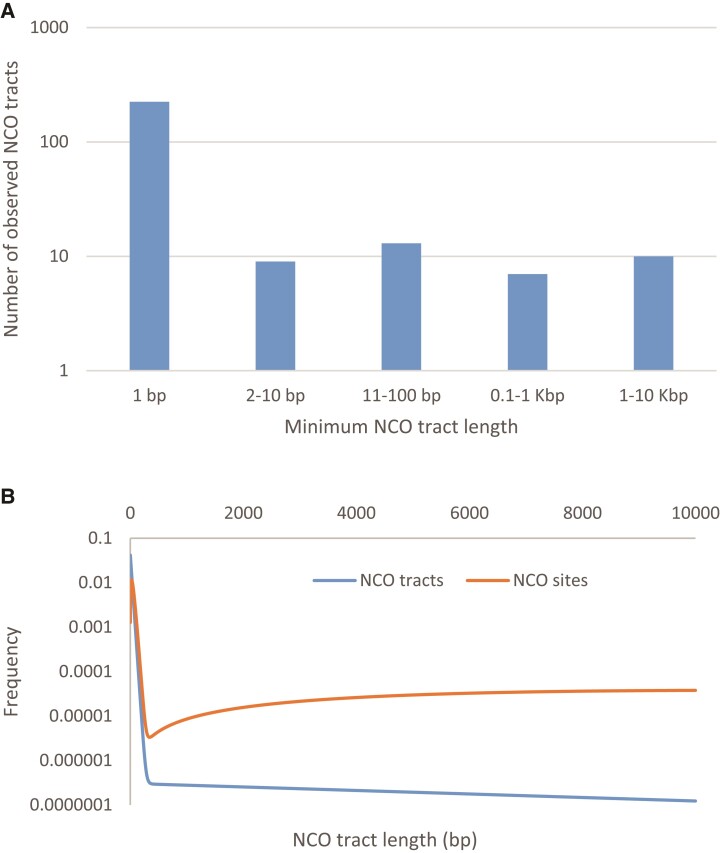
Distribution of NCO tract lengths, for actual data and best-fit model. (*A*) Minimum inferred lengths of observed NCO tracts shorter than 10 kb. (*B*) Distribution of NCO tract lengths for the best-fit mixture of two geometric distributions model (in blue), and weighted by tract length (in orange).

We used a maximum-likelihood approach for estimating the NCO tract length distribution from the observed patterns of converted NCO sites. We first assumed a geometric tract length distribution, similar to previous studies (e.g., Hilliker et al. 1994; Gay et al. 2007; Miller et al. 2012; Li et al. 2019). If we confine our analyses to NCO tracts <10 kb in length, we estimate a mean tract length of 309 bp (95% confidence interval (CI) = 290–341 bp). However, we found that our estimate was roughly proportional to the minimum length of the longest NCO tract in our data set. For example, if we only consider NCO tracts <5 kb in length, the estimate is 182 bp (95% CI = 173–201 bp), or for tracts <1 kb, the mean length estimate is 58 bp (95% CI = 48–71 bp). This is in part because the geometric distribution does not fit the data well. In particular, while most NCO sites are consistent with a short (i.e., < 100 bp) tract length, there is a tail of longer NCO tracts that must be kilobases long (fig. 6*A*).

We next considered a more general scenario where NCO tracts shorter than 10 kb have lengths that are modeled as a mixture of two truncated geometric distributions. This would be appropriate if NCO recombination could occur through two separate molecular pathways, each of which produced tracts whose length followed geometric distributions. Our maximum likelihood estimate had 99.8% of NCO tracts following a distribution with a mean length of 24 bp (95% CI = 18–31 bp), while the remaining NCO tracts had a mean length of 4.3 kb (95% CI = 2.6–4.9 kb). Under this best-fit model, just 1.6% of NCO tracts are longer than 100 bp, but these longer tracts account for 31.6% of all sites that are converted by NCO recombination (see blue and orange curves in fig. 6*B*, respectively).

## Discussion

Pedigree-based studies of recombination, while common in previous decades due to technological and computational limitations, have been mostly superseded now by studies that indirectly estimate recombination rates from patterns of LD (e.g., Beeson et al. 2019; Dreissig et al. 2019; Jones et al. 2019; Robinson et al. 2019; Shanfelter et al. 2019; Spence and Song 2019; Xue et al. 2019; Apuli et al. 2020; Pfeifer 2020; Schield et al 2020; Schwarzkopf et al. 2020). We argue though that despite the substantial amount of time and effort required to conduct pedigree-based studies, they can provide invaluable information that is inaccessible by other methods. LD-based recombination estimates, by their nature, are averages across time and individuals, are influenced by any evolutionary force that affects patterns of genetic variation (e.g., changes in population size, migration, admixture, natural selection, etc.), and require assumptions about the effective population size to be converted into an actual per generation rate. They cannot provide any information on sex-specific differences in CO rates, nor are they very informative about NCO recombination. For example, while baboon pyrho estimates are, on average, slightly elevated in sub-telomeric regions, this obscures the observation that male COs are 10–15 times more prevalent than female COs in the distal 5 Mb of each chromosome arm. Human pedigree studies show the same general pattern (Broman et al. 1998; Kong et al. 2002), but the sex bias is <2-fold in humans.

One ancillary benefit of our pedigree-based examination of recombination in baboons is that it helped provide some independent information on the quality of the existing Panubis1.0 genome assembly. Panubis1.0 utilized a combination of Illumina short-read, Oxford Nanopore long-read, 10× Genomics linked-read, Bionano optical map, and Hi-C sequence data to create an assembly with N50 contig size of 1.46 Mb and single scaffolds that span each of the autosomes (Batra et al. 2020). The Hi-C data in particular enabled Panubis1.0 to be a truly de novo genome assembly, unlike the previous reference-guided baboon assembly (Rogers et al. 2019). However, there is some concern that Hi-C-based scaffolding is susceptible to the incorrect orientation of contigs, leading to inversion errors in the resulting assembly (e.g., Burton et al. 2013). Here, traditional linkage analyses enabled us to identify more than 20 likely assembly errors, most of which were putative inversions (supplementary table S3, Supplementary material online). This suggests that caution should be taken in accepting Hi-C-based scaffolding without the presence of orthogonal sources of corroborating data.

Evidence for an inversion assembly error in linkage data comes from the presence of three closely spaced COs in one or more individuals (Broman et al. 1998, 2003; fig. 2). Six of these cases are also consistent with a single CO associated with a long (24–86 kb), nearby NCO tract. While the data that we have cannot rule out either of these explanations, the relative dearth of putative long NCO tracts that are not associated with potential genome assembly errors strongly suggests that most (if not all) of the apparent long baboon NCO tracts are artifacts and not real. Similarly, we speculate that while many long human NCO tracts identified in previous studies (Williams et al. 2015; Halldorsson et al. 2016) are likely to be real (based on evidence of gBGC within them), some of them may actually be spurious and caused by microassembly errors or polymorphic structural variants. It is also likely that some of them represent complex NCO events (with multiple smaller conversion tracts) that are misclassified due to low marker density.

Finally, we note that even after removing all apparent long (e.g., >10 kb) NCO tracts, our data show that a simple geometric model of NCO tract lengths is inappropriate, at least for baboons. While the mixture of two truncated geometric distributions model we considered is somewhat arbitrary, it captures the qualitative observation that most baboon NCO tracts are quite short, but a small minority can be much longer. The extent to which our findings reflect general patterns of NCO recombination is unclear at this time. To date, only two other mammalian species have been studied in depth. Our results are qualitatively similar to the findings of NCO studies in humans (Williams et al. 2015; Halldorsson et al. 2016), but long NCO tracts seem to be much rarer in studies of hybrid mice (Li et al. 2019; Gergelits et al. 2021). Williams et al. (2015) identified three contiguous long NCO tracts >9 kb in length in humans (cf. fig. 5, Williams et al. 2015) that they excluded from their primary analyses. Using a conservative estimate of the mean human NCO tract length of 300 bp (cf. Jeffreys et al. 2001), we find that the probability of observing 3 out of ∼100 tracts longer than 9 kb is ∼0 (i.e., <10^−30^). High-resolution studies in additional species will be needed to better understand how empirical patterns of recombination vary across species, and whether additional molecular models (e.g., a CO pathway without interference) may be necessary to explain the inferred patterns of recombination in large pedigrees.

## Materials and Methods

### Samples, Sequencing, and Variant Calling

All samples for this study are putative olive baboons (*P. anubis*) from the pedigreed baboon colony housed at the Southwest National Primate Research Center (SNPRC). We extracted DNA from blood or tissue samples and sent them to MedGenome, Inc. for sequencing on Illumina HiSeq 4000 and X machines (450 bp mean insert size, 150 bp × 150 bp paired-end sequencing). We generated novel whole-genome sequence data from 23 individuals, generated additional sequence data from several previously published baboon genomes, and combined these with data from previous studies (Robinson et al. 2019; Wu et al. 2020) to obtain a final data set that included 66 baboons with a median of 35.6× depth of coverage. These samples are included in two large pedigrees (fig. 1) as well as in a panel of 36 unrelated olive baboons. SRA accession numbers for all sequences used in this study are presented in supplementary table S4, Supplementary material online and archived in NCBI BioProject PRJNA433868.

We used a pipeline adapted from the Best Practices workflow for the Genome Analysis Toolkit v3 (GATK v3.8-1-0-gf15c1c3ef; McKenna et al., 2010) to generate joint variant call format (VCF) files as follows. For each sample, we first trimmed reads to remove adapter sequence contamination and low-quality bases with TrimGalore v0.6.4 (https://www.bioinformatics.babraham.ac.uk/projects/trim_galore) using the following options: -q 20 --stringency1 --length 50. We then aligned each trimmed read set to the Panubis1.0 genome assembly (GCA_008728515.1; Batra et al. 2020) using BWA MEM v0.7.17 (Li 2013) before marking duplicate reads with Picard v2.21.3 (https://broadinstitute.github.io/picard) and then genotyping with GATK HaplotypeCaller. We then produced a joint call set with GATK GenotypeGVCFs, followed by GATK LeftAlignAndTrimVariants, before applying filters. Specifically, we excluded sites in soft-masked regions of the genome assembly, which correspond to repetitive and low complexity regions identified with WindowMasker (Morgulis et al., 2006), plus variants that were not bi-allelic SNPs. We also excluded genotypes with genotype quality (GQ) score <30, and sites with excess heterozygosity (defined as sites where the number of heterozygotes is >3.5 standard deviations above random mating expectations). For the last criterion, we are aware that the 66 samples are not all unrelated, but since relatedness (and population structure) generally leads to less heterozygosity than random mating expectations, our approach is conservative.

### Pedigree-based Identification of CO Events

We utilized the single-nucleotide variants in the call set described above to identify recombination events from the meioses involving 10173, 12242, 9841, 1X2816, 1X4519, and their offspring (20 paternal meioses and 17 maternal meioses in total). Note that each sub-pedigree had a minimum of five offspring.

For target meiosis, we first filtered the data to include only “informative” sites where the parentally transmitted allele could be directly inferred. For example, when trying to identify paternal recombination events in 16517, we require the sire’s (10173) genotype to be heterozygous and the dam’s (12242) genotype to be homozygous. That way, the maternally transmitted allele is known and the paternally transmitted allele must be the other allele in 16517’s genotype. While the parental genome is unphased, it is straightforward to infer haplotypic phase by examining the patterns of alleles transmitted by the parent and to identify potential recombination events by switches in which haplotype is inherited in each of the offspring (Coop et al. 2008). We employed additional filters by requiring genotype calls in all of a pedigree’s offspring, and by removing the five informative sites nearest to the ends of each chromosome.

We started by identifying all switches in transmitted haplotype that could be parsimoniously explained by a single CO (supplementary table S1, Supplementary material online). We then manually examined all intervals (i.e., regions between consecutive informative markers) where we inferred the occurrence of two COs (in two separate meioses). Four of these intervals were long (e.g., >60 kb), not near chromosome ends (i.e., at least 2 Mb away), and involved a sub-pedigree with at least seven offspring (i.e., with parent 10173, 1X2816, or 1X4519). For these, the evidence is quite strong that there were in fact two COs (rather than a genome assembly error or >2 COs). An additional eight intervals are shorter (2–30 kb) and/or involve smaller sub-pedigrees with only five offspring. We have labeled these as “provisional” COs, listed them in the “Provisional_COs” sheet in supplementary table S1, Supplementary material online, and included them for estimating the total genetic map length.

### Pedigree-based Identification of Genome Assembly Errors

We identified several unusual patterns in our CO data that are suggestive of either genome assembly errors or polymorphic chromosomal rearrangements (fig. 2). For example, three closely spaced COs in a single meiosis are extremely unlikely due to CO interference (Muller 1916), but could easily arise through the combination of a single CO plus an inversion (Broman et al. 1998, 2003). We hypothesized that potential breakpoints are likely to occur in-between assembled contigs, and classified these synteny breaks as putative inversions (three COs within 10 Mb in the same meiosis), misplaced contigs (two closely linked COs occurring at the same locations in multiple individuals), translocations (misplaced contigs where the correct genomic location could be inferred), or single breaks of synteny (multiple COs from different meioses mapped to the same small region) (supplementary table S2, Supplementary material online). The genomic location of contig breaks within hypothesized breakpoints is shown in parentheses in the second and third columns of supplementary table S2, Supplementary material online. Note that if contig breaks happened at random relative to the locations of synteny breaks, the expected overlap is ∼1.2. Instead, we observe 31 contig breaks at the synteny breaks in supplementary table S2, Supplementary material online. Finally, we acknowledge that the boundary between single synteny breaks and “provisional crossovers” (as defined above) is somewhat arbitrary. In supplementary table S2, Supplementary material online, we eventually included two single synteny breaks: one (on chromosome 20) where more than five COs could be localized to a 44 kb region, and the other where two COs could be localized to a 1 kb region on chromosome 13.

We then modified the Panubis1.0 genome assembly to account for all of the putative assembly errors listed in supplementary table S2, Supplementary material online where the breakpoint(s) can be localized to a 5 kb or smaller region. The exact changes made to Panubis1.0 are listed in supplementary table S4, Supplementary material online. Of note, there is overwhelming evidence for a synteny break near position 13004575 on chromosome 20, with at least 6 COs mapping to a 40 kb interval. However, the correct order and orientation for chromosome 20 scaffolds is unclear from the data that we have. We have opted to label the two fragments as chromosomes 20A and 20B, with the assumption that future genetic studies will be needed to resolve outstanding baboon assembly issues.

### LD-based Recombination Estimates

We used pyrho v0.1.5 (Spence and Song 2019) to estimate local recombination rates from patterns of LD in a panel of 36 unrelated olive baboons. Recombination rate inference with pyrho requires a demographic model as input. We used SMC++ v1.15.4.dev16 + g72ea2e2.d20200621 (Terhorst et al., 2017) to infer a demographic model from the 36 unrelated baboons prior to running pyrho, as recommended in the pyrho documentation. First, we applied additional hard filters to the variant call set to minimize the inclusion of errors. Specifically, we excluded genotypes with GQ <40 and with read depth <8. We also applied a series of filters based on GATK hard-filtering recommendations to exclude low confidence variants (QUAL <30.0, QD <2.0, FS >60.0, MQ <40.0, MQRankSum less than −12.5, ReadPosRankSum less than −8.0, SOR >3.0, ExcHet <0.05), leaving 14.4 million variants in total. Note that low complexity and repetitive sequences were previously excluded as described above (see Samples, Sequencing, and Variant Calling), and their coordinates were used as the “mask” when converting VCF files to SMC++ input files. Using the “estimate” function of SMC++, we then inferred the demographic history from all 36 individuals with a random subset of ten individuals designated as the “distinguished” lineages. In our data set, reference and nonreference alleles are polarized with respect to the underlying reference sequence, which is from an olive baboon (Batra et al., 2020), and thus do not reflect ancestral/derived allele states. We therefore set the polarization parameter (−*p*) to 0.5 when running “estimate” to indicate that the ancestral state is not known, as recommended by the developers. Results obtained with and without invoking this polarization parameter were effectively identical (not shown). We assumed a mutation rate of 5.7×10^−9^ per site per generation and a generation time of 11 years (Wu et al., 2020), and used default settings for all other parameters.

To confirm that our demographic inference is robust despite including genomic regions putatively under selection (see Johri et al., 2021), we repeated the demographic inference with protein-coding sequences masked. Excluding protein-coding regions had little impact on the inferred demographic history (supplementary fig. 2*A*, Supplementary material online), and we therefore used the model inferred with the original mask (containing repeats only) for all analyses. To assess the goodness of fit for our demographic model, we used dadi v2.1.1 (Gutenkunst et al. 2009) to generate the expected distribution of allele frequencies, or site frequency spectrum (SFS), under the demographic model produced by SMC++ (supplementary fig. 2*B*, Supplementary material online), following the method of Beichman et al. (2017). Compared with the observed SFS, the expected SFS shows a slight excess of singletons and an excess of intermediate frequency alleles. Un-modeled complexities, such as population structure or gene flow, may account for these differences; olive and yellow baboons (*Papio cynocephalus*) have a complex history of admixture (Wall et al., 2016; Rogers et al., 2019), and continue to hybridize naturally in contact zones in Africa (Wall et al., 2016; Vilgalys et al., 2021).

We then followed the pyrho documentation to incorporate the demographic model from SMC++ to infer local recombination rates from the 36 unrelated baboons. We applied two additional filters before running pyrho: we excluded singletons, which are uninformative for LD-based recombination rate inference, and we thinned variants so that no two SNPs were closer than 10 bp, leaving 10.4 million variants in total. We used default settings for all other parameters, except where noted. As above, we assumed a mutation rate of 5.7×10^−9^ per site per generation (Wu et al., 2020). To handle the large number of haplotypes in our dataset (*n* = 2 × 36 = 72), we enabled the Moran approximation method when generating the lookup table with: --approx --moran_pop_size 98. To determine the optimal values for the window size and block penalty (smoothing) hyperparameters, we followed the pyrho documentation and used the ranking strategy from Spence and Song (2019) to determine that a block penalty of 10 and window size of 75 were predicted to yield the most accurate rate estimates. After manual inspection of the output, we removed the pyrho rate estimate for a single interval on chromosome 19 (approximate positions 24.92–25.02 Mb) where the estimated genetic map length was ∼102 cM. There is no evidence for any COs near this region, so we deemed the extremely large estimate to be unreliable.

pyrho and other LD-based methods most naturally estimate the population scaled recombination parameter, *ρ* (= 4*Nr*, where *N* is the effective population size and *r* is the recombination rate per generation). So, conversion of these values to actual per generation recombination rate estimates requires assumptions about other fundamental parameters such as *N* and/or the mutation rate. With the assumptions described above, the pyrho estimated total genetic map length was several times shorter than the sex-averaged CO-based genetic map length. To make these results more compatible with each other, we rescaled the pyrho values to have the same total autosomal map length (2,293 cM) as estimated from COs by multiplying all pyrho-based estimates by the constant 5.577. This preserves local patterns of recombination rate variability while acknowledging the large uncertainties in estimating historical population sizes.

### Identifying NCO Recombination

NCO recombination can be identified from pedigree data in an analogous way as CO identification. Specifically, single NCO tracts show up as two very tightly linked COs in a single individual, or equivalently as one or more closely linked sites where an offspring inherits one parental haplotype, surrounded on both sides by much larger regions where the offspring inherits the other parental haplotype. Finally, we arbitrarily fixed the maximum NCO tract length size as 10 kb and analyzed apparent larger NCO tracts separately (see Results).

Pedigree 1 contained three offspring (32043, 32849, and 33863) of the second-generation individuals used for estimating NCO rates (fig. 1). For all sites contained in putative NCO tracts inherited by the parents of these offspring (19181 and 19348), we checked for Mendelian inconsistencies across the whole pedigree as a limited way to test whether putative NCO tracts might be caused by sequencing/genotyping errors in the offspring. (We did not find any.) To reduce the effect of potential genotyping errors in the parent, we excluded sites with more than two segregating alleles, as well as all apparent NCO tracts that are shared across multiple half or full siblings.

### NCO Tract Length Distribution

We use an approximate maximum-likelihood approach to estimating the probability of the data as a function of NCO tract length distribution parameters. Here the data consist of the pattern of which informative sites are converted (or not converted) for each autosome of each meiosis. We assume that both the probability of NCO recombination and the NCO tract length distribution do not change across base pairs or meioses, and fix the former at 7.52 × 10^−6^ per base pair per generation, as estimated below. Without loss of generality, we assume that NCO tracts are initiated at a certain base pair and then continue along the chromosome 5′ to 3′ until they end.

Suppose *D*(Λ) is a specific NCO tract length distribution governed by parameter(s) Λ. If *m*(Λ) is the mean tract length given Λ, then the per base pair probability of initiation of an NCO tract of length *k* is

$$f_{k}(\Lambda )=\frac{7.52\times 10^{-6}\mathrm{Pr}(k|\Lambda )}{m(\Lambda )}$$

per meiosis. For a specific NCO tract, define {*o_k_*} as the number of *k*-mers (i.e., *k* consecutive base pairs) that overlap all of the informative sites converted in the tract and no others. Then the probability of observing an NCO tract is $\sum_{k}\;f_{k}(\Lambda )o_{k}$. Similarly, define {*e_k_*} as the number of *k*-mers (across a chromosome) that must be excluded because they overlap with a nonconverted site. The probability of observing these nonconverted sites is $\prod_{k}\;(1-f_{k}(\Lambda ){)}^{e_{k}}\approx e^{-\sum_{k}\;f_{k}(\Lambda )e_{k}}$. Finally, the likelihood of the full data is then the product of the separate likelihoods of observing each of the observed NCO tracts multiplied by the products of all of the probabilities of not converting the nonconverted sites, across all meioses and autosomes.

Our analyses of actual data treated each contiguous NCO tract as separate, even if it was part of a complex NCO event, and arbitrarily required tract lengths to be 10 kb or shorter. This length cutoff was chosen based on the observation that most (nine out of ten) apparent NCO tracts between 10 and 100 kb are consistent with being caused by microassembly errors (cf. table 1). This led to a total of 263 NCO tracts that we had information for. We first considered a geometric distribution for NCO tract lengths, requiring the mean length to be an integer. Confidence intervals were obtained using the standard asymptotic maximum likelihood assumptions. Next, we considered a mixture of two truncated geometric distributions, parameterized by Λ = (*α*, *m*_1_, *m*_2_), where *m*_1_ and *m*_2_ are the means of the two corresponding nontruncated distributions, and *α* is the proportion of tracts that have mean *m*_1_. We then calculated the likelihood of the data over a grid of parameter values, where *α* varied from 0 to 1 in increments of 0.001 and *m*_1_ and *m*_2_ varied from 10 to 100,000 for all integers with two significant digits in this range. After obtaining the maximum likelihood estimate, we created profile likelihood curves for *m*_1_ and *m*_2_ to estimate approximate confidence intervals.

## Supplementary Material

Supplementary data are available at *Genome Biology and Evolution* online.

## Supplementary Material

evac040_Supplementary_Data

## Data Availability

All sequence data used in this study have been deposited in the Sequence Read Archive under NCBI BioProject PRJNA433868 with accession numbers given in supplementary table S5, Supplementary material online. The VCF files and pyrho genetic map are publicly available at https://doi.org/10.7272/Q6HH6H9D. The updated baboon genome assembly Panubis1.1 has been deposited at DDBJ/ENA/GenBankunder the accession VSMJ00000000. The version describedin this paper is version VSMJ02000000.
