# Supplementary material for: High-Resolution Estimates of Crossover and Noncrossover Recombination from a Captive Baboon Colony
Source: Genome Biol Evol. 2022 Mar 24;14(4):evac040. doi: 10.1093/gbe/evac040 (PMC9048888; doi:10.1093/gbe/evac040)
Supplement: evac040_Supplementary_Data [file evac040_supplementary_data.zip › Babped_supp1.pdf]

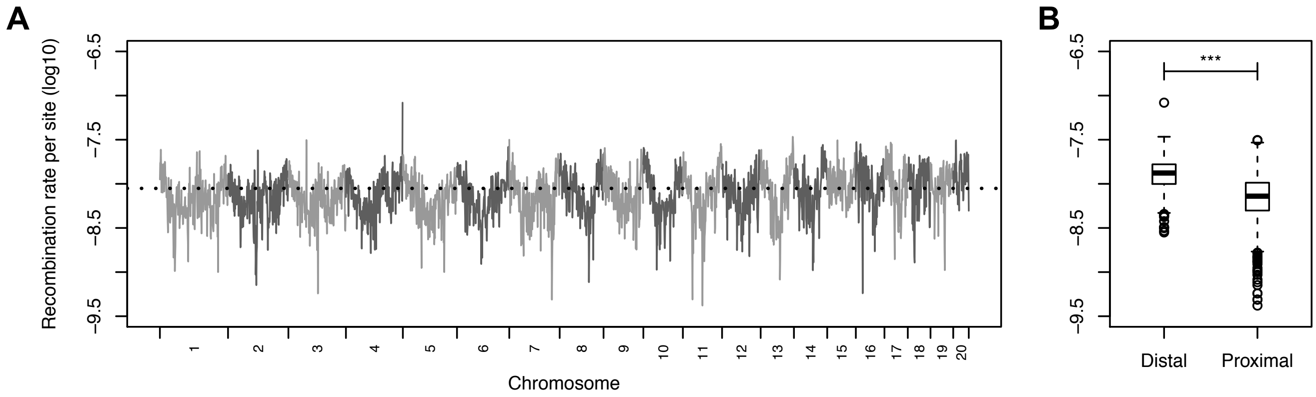

Figure S1. LD-based estimates of recombination using pyrho. (A) Average recombination rate estimates in non-overlapping 1 Mb windows across the genome. (B) Boxplots showing the significant difference in pyrho estimates between proximal (i.e., > 10 Mb from chromosome ends) and distal (<10 Mb from chromosome ends) windows.

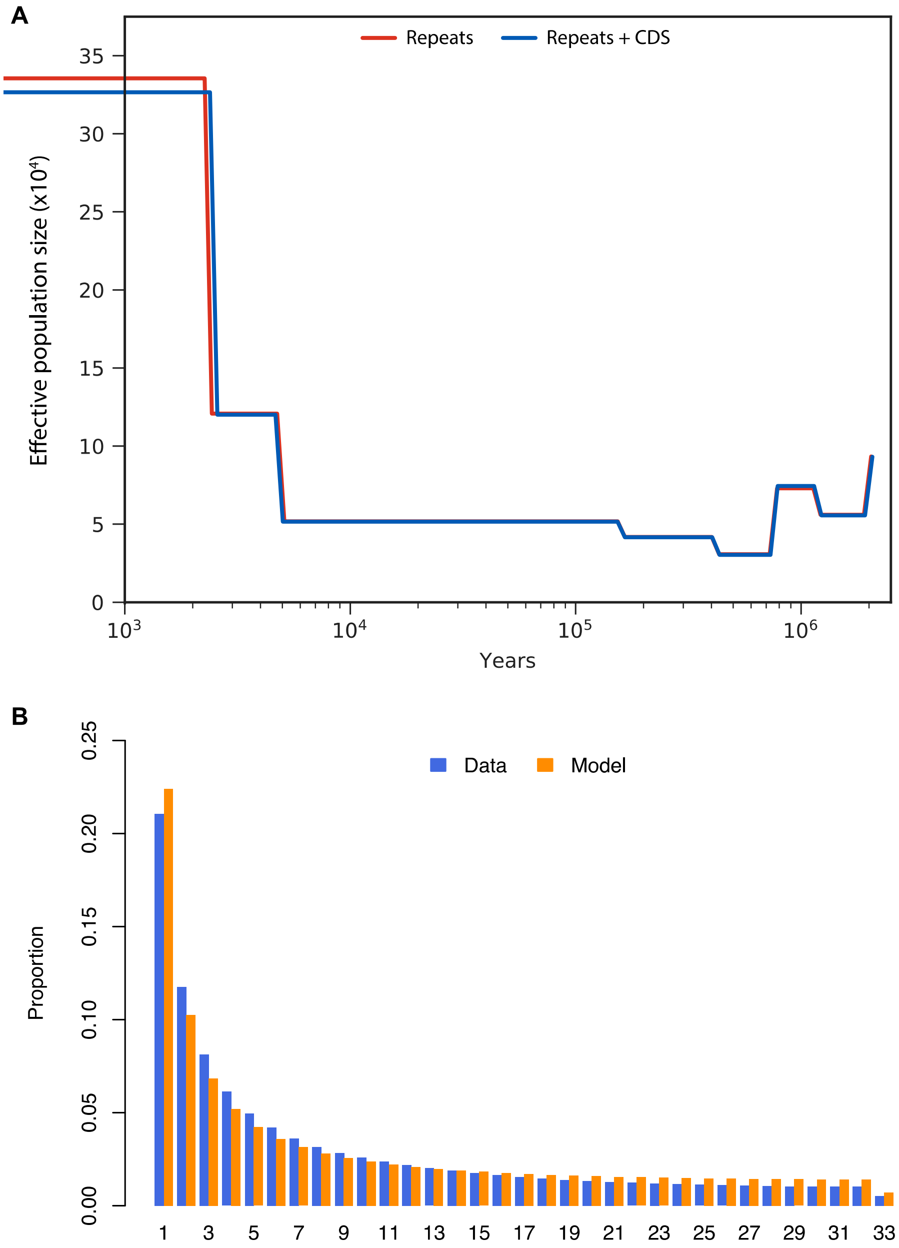

Figure S2. Demographic model inferred by SMC++ and model fit. A) Demographic history inferred from 36 unrelated olive baboons with and without masking protein-coding sequences ("CDS") in addition to repetitive sequences. The trajectories are extremely similar, suggesting the inference is not strongly affected by including genomic regions subject to selection. The "Repeats" model was used for all analyses. B) Barplot showing the fit of the folded proportional site frequency spectrum (SFS) expected under the demographic model inferred with SMC++ ("Model") compared to the observed SFS ("Data"). The expected SFS was generated with ∂a∂i.

Table S2. List of potential genome assembly errors identified from patterns of crossovers in pedigrees

Chr	Breakpoint 1[1]	Breakpoint 2[1]	Parent(s)	Offspring	Type	pyrho confirmation?
4	128908402-128926502 (128926072)	129083010-129133287 (129115279)	9841	15444	Inversion	Yes
5	87107633-87174522 (87174124)	87763274-87834276 (87832318)	1X4519	9128	Inversion	No
6	19187190-19205515 (19204753)	19418591-19427887 (19426981)	10173	18385	Inversion	Yes
7	152346-183891 (162649)	578048-619612 (553186)	10173	15444	Inversion	Inconclusive
7	2108730-2110031 (2109398)	2181650-2183146 (2182558)	>3	>8	Misplaced contig	Yes
7	96278371-96282033 (96279505)	96803605-96811375 (96805194)	1X4519, 1X2816	6955, 7625	Inversion	Yes
7	100778767-100780278	105498863-105705880 (105499543)	1X4519	10987	Inversion	No
7	114482280-114482897	115014663-115030265 (115020065)	1X4519	10987	Inversion	No
7	154337477-154346856 (154340366)	156816763-156827272 (156821679)	1X2816, 9841	10987, 19348	Inversion	No
7	156818082-156827354 (156821780)	157535325-157538265	10173	26988, 28246	Misplaced contig	Yes
8	43476352-43564391 (43550211)	43635387-43639241	10173, 1X2816	>4	Misplaced contig	Yes
11	39803972-39849254 (39808739)	40005564-40036643 (40010327)	1X2816	10489	Inversion	Yes
12	101260717-101277410 (101270326)	106479800-106525157 (106481018)	12242	16517, 17903, 28246	Inversion	Yes
13	7037503-7174125	7552177-7575804	1X2816	8307	Inversion	No

	(7059365)	(7555231)				
13	76945690-77038672	77379993-77385849	1X2816	16517	Inversion	N/A
13	103472993-103476718 (103476237)	104948032-104950546 (104948753)	1X2816	7311	Inversion	No
13	103793201-103794244		10173	15444, 17199	Synteny break	Yes
16	52176980-52180999 (52179410)	52308031-52316803 (52308689)	10173, 1X2816	>5	Translocation	Yes[2]
20	3112639-3115037 (3114115)	4152939-4154479 (4153923)	10173	17903, 28246	Inversion	No
20	9060715-9068752	9382803-9391671	10173	18385	Inversion	N/A
20	13003097-13047006 (13004575)		10173, 1X2816	>5	Synteny break	N/A
20	37314503-37350014	37712955-37718236	10173	26988	Inversion	N/A

[1] Region where breakpoint can be localized, with coordinates of gap between contigs in parentheses when available
[2] Region is likely syntenic with the end of chromosome 16 (after approximate position 89.67 Mb)

Table S4. Comparison of syntenic regions of the Panubis1.0 and Panubis1.1 assemblies in their respective coordinates. All chromosomes (and scaffolds) not listed below are identical between Panubis1.0 and Panubis1.1. Note that slight misalignment of Panubis1.0 coordinates (e.g., on chr16) is needed to account for the 100 N's needed to represent gaps of unknown length.

Panubis1.0	Panubis1.1	Notes
chr4:1-128926121	chr4:1-128926121	No change
chr4:128926122-129115228	chr4:129115228-128926122	Inverted in Panubis1.1
chr4:129115229-182120902	chr4:129115229-182120902	No change
chr5:1-87174124	chr5:1-87174124	No change
chr5:87174125-87832319	chr5:87832319-87174125	Inverted in Panubis1.1
chr5:87832320-173900761	chr5:87832320-173900761	No change
chr6:1-19204753	chr6:1-19204753	No change
chr6:19204754-19426982	chr6:19426982-19204754	Inverted in Panubis1.1
chr6:19426983-167138247	chr6:19426983-167138247	No change
chr7:1-162649	chr7:1-162649	No change
chr7:162650-553187	chr7:553187-162650	Inverted in Panubis1.1
chr7:553188-2109398	chr7:553188-2109398	No change
chr7:2109399-2182659	scaffoldA:1-73161	Misplaced contig
chr7:2182660-96279505	chr7:2109399-96206244	No change
chr7:96279506-96805195	chr7:96731934-96206245	Inverted in Panubis1.1
chr7:96805196-100778766	chr7:96206246-100705505	No change
chr7:100778767-100780278	scaffoldB:1-1512	Unplaced sequence
chr7:100780279-105499544	chr7:105424771-100705506	Inverted in Panubis1.1
chr7:105499545-114482279	chr7:105424772-114407506	No change
chr7:114482280-114482897	scaffoldC:1-618	Unplaced sequence
chr7:114482898-115020066	chr7:114944675-114407507	Inverted in Panubis1.1
chr7:115020067-154340366	chr7:114944676-154264975	No change
chr7:154340367-156821680	chr7:156746289-154264976	Inverted in Panubis1.1
chr7:156821681-157538265	scaffoldD:1-716485	Misplaced contig
chr7:157538266-161768468	chr7:156746390-160976592	No change
chr8:1-43550211	chr8:1-43550211	No change
chr8:43550212-43639241	scaffoldE:1-89030	Misplaced contig
chr8:43639242-140274886	chr8:43550212-140185856	No change
chr11:1-39808739	chr11:1-39808739	No change
chr11:39808740-40010328	chr11:40010328-39808740	Inverted in Panubis1.1
chr11:40010329-125913696	chr11:40010329-125913696	No change
chr12:1-101270326	chr12:1-101270326	No change

chr12:101270327-106481019	chr12:106481019-101270327	Inverted in Panubis1.1
chr12:106481020-123343450	chr12:106481020-123343450	No change
chr13:1-7059365	chr13:1-7059365	No change
chr13:7059366-7555232	chr13:7555232-7059366	Inverted in Panubis1.1
chr13:7555233-103476237	chr13:7555233-103476237	No change
chr13:103476238-104948754	chr13:103476238-104948754	Inverted in Panubis1.1
chr13:104948755-106849001	chr13:104948755-106849001	No change
chr16:1-52179410	chr16:1-52179410	No change
chr16:52179311-52308690	chr16:91054814-91184193	Translocation in Panubis1.1
chr16:52308791-91184193	chr16:52179411-91054813	Translocation in Panubis1.1
chr20:1-3114115	chr20A:1-3114115	No change
chr20:3114116-4153924	chr20A:4153924-3114116	Inverted in Panubis1.1
chr20:4153925-13004526	chr20A:4153925-13004526	No change
chr20:13004627-50021108	chr20B:1-37016482	See below

We found strong evidence for a synteny break at chr20:13004527, but could not identify the correct orientation for the two chromosome fragments. We have opted to place them in separate “chromosomes” for now, with the hope that future genetic studies can elucidate the proper order and orientation of these sequences.
